# Acute effects of intermittent hypoxia–hyperoxia exposure on cardiovascular autonomic function and blood pressure in sedentary older adults: A pilot randomized controlled trial

**DOI:** 10.1371/journal.pone.0350802

**Published:** 2026-06-26

**Authors:** Arturo Ladriñán-Maestro, Alberto Sánchez-Sierra, Oualid Elfoukahi, Óscar Martín-Moreno, Lucía Ladriñán-Maestro, Jorge Sánchez-Infante

**Affiliations:** 1 Faculty of Physiotherapy and Nursing of Toledo, Universidad de Castilla-La Mancha, Toledo, Spain; 2 Physiotherapy Research Group of Toledo (GIFTO), Faculty of Physiotherapy and Nursing, Universidad de Castilla-La Mancha, Toledo, Spain; 3 Faculté des Sciences de la Rééducation et des Techniques de Santé, Universiapolis Université Internationale d’Agadir, Agadir, Morocco; 4 Physiotherapy Department, Nursing Home Montes de Toledo, Manzaneque, Spain; 5 Rehabilitation Department, Toledo University Hospital, Toledo, Spain; 6 Institute of Health and Sport Sciences, Faculty of Health Sciences, Universidad Francisco de Vitoria, Madrid, Spain; Cairo University Kasr Alainy Faculty of Medicine, EGYPT

## Abstract

**Background:**

The aim of this study was to evaluate the acute effects of a single session of intermittent hypoxia–hyperoxia exposure on cardiovascular autonomic regulation, blood pressure, and arterial oxygen saturation in sedentary older adults.

**Methods:**

A double-blind randomized controlled trial with pre- and post-intervention assessment was conducted in a residential care physiotherapy setting. Sixteen sedentary older adults were randomly assigned to an experimental group (n = 8) or a control group (n = 8). The experimental group received one session of intermittent hypoxia–hyperoxia exposure consisting of six cycles of hypoxia (FiO₂ 10–14% for 5 minutes) followed by hyperoxia (FiO₂ 30–40% for 3 minutes), administered via a hypoxic generator. The control group received a sham intervention consisting of normoxic breathing (FiO₂ 21%). Heart rate variability (SDNN, RMSSD, LF/HF), systolic and diastolic blood pressure, heart rate, and arterial oxygen saturation were measured at baseline and immediately after the intervention.

**Results:**

Compared with the control group, the experimental group showed significant increases in SDNN and RMSSD and a significant reduction in the LF/HF ratio (p < 0.01), indicating improved autonomic regulation. Systolic and diastolic blood pressure and heart rate decreased significantly in the experimental group compared with controls (p < 0.01). Arterial oxygen saturation showed a small but significant increase in the experimental group (p < 0.01). No adverse events or withdrawals were reported.

**Conclusions:**

In this pilot randomized controlled trial, a single session of intermittent hypoxia–hyperoxia exposure was associated with acute changes in heart rate variability indices and blood pressure in sedentary older adults. These preliminary findings should be interpreted cautiously and require confirmation in larger, adequately powered trials.

## 1. Introduction

Oxygen delivery is vital for energy production, recovery from physical exertion and overall human survival. During prolonged and/or intense physical exercise, the body’s energy demands increase, which is directly linked to a greater oxygen requirement. The 2019 Nobel Prize in Medicine demonstrated that working in hypoxia, through the modulation carried out by HIF-1, can activate over 300 genes associated with improvements in inflammatory, cognitive, cardiorespiratory and nervous system functions, among others [1]. Intermittent hypoxia-hyperoxia exposure (IHHE) is a non-invasive and non-pharmacological technique that subjects individuals to a controlled respiratory environment with a reduced oxygen fraction. This well-tolerated therapy consists of alternating and repetitive cycles of normobaric hypoxia and hyperoxia, representing an intervention method applicable to both healthy and clinical populations [2,3]. It is administered via a facial mask connected to a hypoxic generator device, with oxygen saturation continuously monitored by specialized software linked to a pulse oximeter [4].

Numerous studies have demonstrated the efficacy of IHHE in diverse clinical populations, including geriatric individuals [4,5] and patients with metabolic syndrome [6]. These studies have consistently shown the safety of its application [5,6] and its benefits for cardiovascular health, inflammatory status and exercise tolerance [4–6]. Furthermore, IHHE appears to play a protective role in the central nervous system against neurodegenerative diseases such as Alzheimer’s and Parkinson’s [7,8]. These changes result in improvements in spatial memory, myocardial function, a reduction in inflammatory status and enhanced aerobic capacity [4,5].

The global increase in the population aged over 65, coupled with rising sedentary behavior, a higher number of comorbidities and polypharmacy is an undeniable fact [9,10]. This makes it increasingly important to integrate non-pharmacological approaches into the therapeutic management of older adults [11]. Given the previously demonstrated benefits, hypoxia-hyperoxia exposure represents a promising intervention for sedentary older adults. However, the impact of this combined therapy on other critical variables such as the sympathetic-parasympathetic balance, blood pressure or arterial oxygen saturation (SpO₂) (all factors of significant importance for the health and quality of life of geriatric patients) remains less explored. Therefore, this study aimed to objectively evaluate the acute effects of IHHE on heart rate variability (HRV), blood pressure and SpO₂ in sedentary older adults.

## 2. Methods

### 2.1 Study design

This study employed a randomized, double-blind clinical trial design, conducted at the Physiotherapy Department of Residencial Montes de Toledo (Manzaneque, Spain), following the Consolidated Standards of Reporting Trials (CONSORT) guidelines [12]. Written informed consent was obtained from all participants. This study was approved by the Research Ethics Committee of the Complejo Hospitalario Universitario de Toledo (registry number: 1071) and registered at ClinicalTrials.gov (NCT06686238).

### 2.2 Participants

A total of sixteen older adults participated in the study. Participants were recruited between 5 December 2024 and 26 December 2024. Using randomization.com software, they were randomly assigned to either the EG, which received an IHHE session, or the CG, which received a sham IHHE application. Participants and outcome assessors were blinded to group allocation. Due to the nature of the intervention, the therapist administering the protocol was not blinded; however, this individual was not involved in outcome assessment or statistical analysis. Inclusion criteria for participants were: being over 60 years old, having no prior experience with hypoxic training, and engaging in less than 150 minutes of physical activity per week. Exclusion criteria included: any pathology that prevents the subject from being independent in walking and functionality, impaired cognitive abilities, pulmonary hypertension, decompensated heart or respiratory disease, and a current or previous history of cancer. The sample size was determined using G*Power Software (3.1.9.2), based on HRV data from a previous study [13]. A power analysis was conducted with an alpha error of 0.05, a beta error of 0.2, and a medium effect size (f = 0.25, or ηp² = 0.06). A 20% estimated dropout rate was considered due to the study design. Therefore, a total sample size of 16 participants, divided into two groups (n = 8 each), was determined.

### 2.3 Intervention

#### 2.3.1 Intermittent hypoxic-hyperoxic exposure.

The EG underwent a single session of IHHE using the MITOVIT® Hypoxic Training System (COMMIT GmbH, Salzgitter, Germany). The session consisted of six alternating cycles of hypoxia and hyperoxia, chosen based on prior studies demonstrating safety and efficacy in older adults. During the hypoxic phase, participants breathed air with a fraction of inspired oxygen (FiO₂) ranging from 10–14% for 5 minutes, targeting an SpO₂ of 85–92%, which is considered safe while eliciting physiological responses associated with hypoxic adaptation. Each hypoxic interval was immediately followed by a 3-minute hyperoxic phase (FiO₂ 30–40%), during which SpO₂ was maintained above 95% to facilitate rapid reoxygenation and mitigate any potential hypoxia-related risks. The transitions between hypoxia and hyperoxia, as well as the precise oxygen delivery, were automatically regulated by the device’s integrated artificial intelligence algorithm, which adjusts FiO₂ in real time based on continuous SpO₂ monitoring. Oxygen saturation and heart rate were continuously recorded throughout the session to ensure participant safety and compliance. Participants were seated comfortably and instructed to breathe normally during all phases, refraining from voluntary physical activity or Valsalva maneuvers. Safety protocols included continuous visual observation by the study personnel and immediate availability of supplemental oxygen [4].

#### 2.3.2 Sham Intermittent hypoxic-hyperoxic exposure.

The CG underwent a single-session protocol using the MITOVIT® Hypoxic Training System (COMMIT GmbH, Salzgitter, Germany). The session consisted of five consecutive cycles of 6 minutes each, during which participants breathed normoxic air (FiO₂ 21%). SpO₂ was continuously monitored throughout the session to ensure safety and verify normoxic conditions. Participants were seated comfortably and instructed to breathe normally during all cycles, refraining from voluntary physical activity or Valsalva maneuvers. Safety protocols included continuous visual observation by the study personnel and immediate availability of supplemental oxygen if required [4].

### 2.4 Outcomes

The primary endpoint of the study was the change in heart rate variability parameters (SDNN, RMSSD, and LF/HF ratio) from baseline to post-intervention. Secondary endpoints included changes in blood pressure, heart rate, and arterial oxygen saturation.

#### 2.4.1 Heart rate variability.

HRV was analyzed using a heart rate monitor (Polar H10; Polar Electro Oy, Kempele, Finland). Cardiac electrical signals were recorded via a chest strap for 5 minutes while participants were in a supine position on a stretcher, in a quiet environment with soft lighting and a comfortable temperature. Participants were instructed to remain still, avoid speaking, and refrain from voluntary movements during the recording. The recorded data were processed using Kubios HRV Analysis Software version 3.1.0 for Windows (Biomedical Signal and Medical Imaging Analysis Group, Department of Applied Physics, University of Kuopio, Finland). The following parameters were extracted: heart rate (HR), RR interval (R-Ri), the standard deviation of all normal-to-normal intervals (SDNN), the sympathovagal balance index calculated as the low-frequency to high-frequency ratio (LF/HF), and the root mean square of successive differences between adjacent normal-to-normal intervals (RMSSD) [14].

#### 2.4.2 Arterial blood pressure.

Both systolic (SBP) and diastolic blood pressure (DBP) were measured using a standard digital sphygmomanometer (Omron HEM-705CP; Omron Healthcare, Inc., Lake Forest, IL). Participants rested in a seated position for 10 minutes prior to the measurements to ensure stabilization of blood pressure. Two consecutive readings were taken, and the average of these values was used for analysis. Measurements were conducted under consistent environmental conditions and participants were instructed to remain relaxed, avoid speaking and keep their arm supported at heart level throughout the procedure [15].

#### 2.4.3 Arterial oxygen saturation.

Arterial oxygen saturation (SpO_2_) was measured using a pulse oximeter (Nonin® 3230; Nonin Medical, Inc., Plymouth, MN, USA). Measurements were conducted for 10 minutes while participants were seated comfortably. The average of the recorded values over the measurement period was used for analysis. Participants were instructed to remain still, avoid speaking and breathe normally throughout the assessment to ensure accurate and reliable readings [16].

### 2.5 Statistical analysis

Statistical analysis was performed using IBM SPSS Statistics v.22.0, with a significance level set at p < 0.05. The Kolmogorov-Smirnov test was employed to assess the normality of each variable, confirming that all variables followed a normal distribution. Descriptive statistics were applied to summarize the demographic characteristics, with results presented as mean ± SD. For the outcome variables, a two-way repeated measures ANOVA was conducted to evaluate the interaction between the Experimental and Control groups across the assessment points (Baseline and Post-treatment). When significant differences were detected, post hoc analyses using Bonferroni multiple comparisons were carried out. Effect sizes for group × time interactions were reported as partial eta squared (ηp²), with values of 0.01, 0.06, and 0.14 interpreted as small, medium, and large effects, respectively [17].

## 3. Results

### 3.1 Demographic data

**Fig 1:**
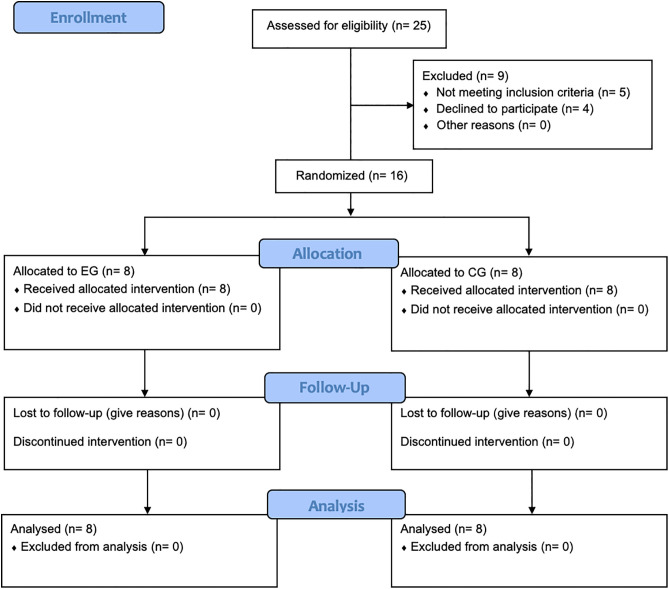
Study flow chart.

A total of sixteen participants were recruited and completed the intervention in December 2024. Participants were randomly assigned to either the Experimental Group (EG) (5 men, 3 women) or the Control Group (CG) (5 men, 3 women). There were no dropouts due to complications or adverse effects, and a CONSORT flow chart is provided (Fig 1). No significant differences in demographic characteristics were observed between the EG and CG (Table 1).

**Table 1:** Demographic characteristics of subject. EG, Experimental Group; CG, Control group.

	EG (n = 8)	CG (n = 8)	p
Sex (male/female)	5/3	5/3	
Age (yrs)	71.50 ± 5.40	72.25 ± 5.04	n.s
Weight (kg)	70.13 ± 7.66	66.63 ± 7.43	n.s
Height (cm)	171.25 ± 6.45	169.00 ± 7.96	n.s

### 3.2 Changes in cardiovascular variables

**Fig 2:**
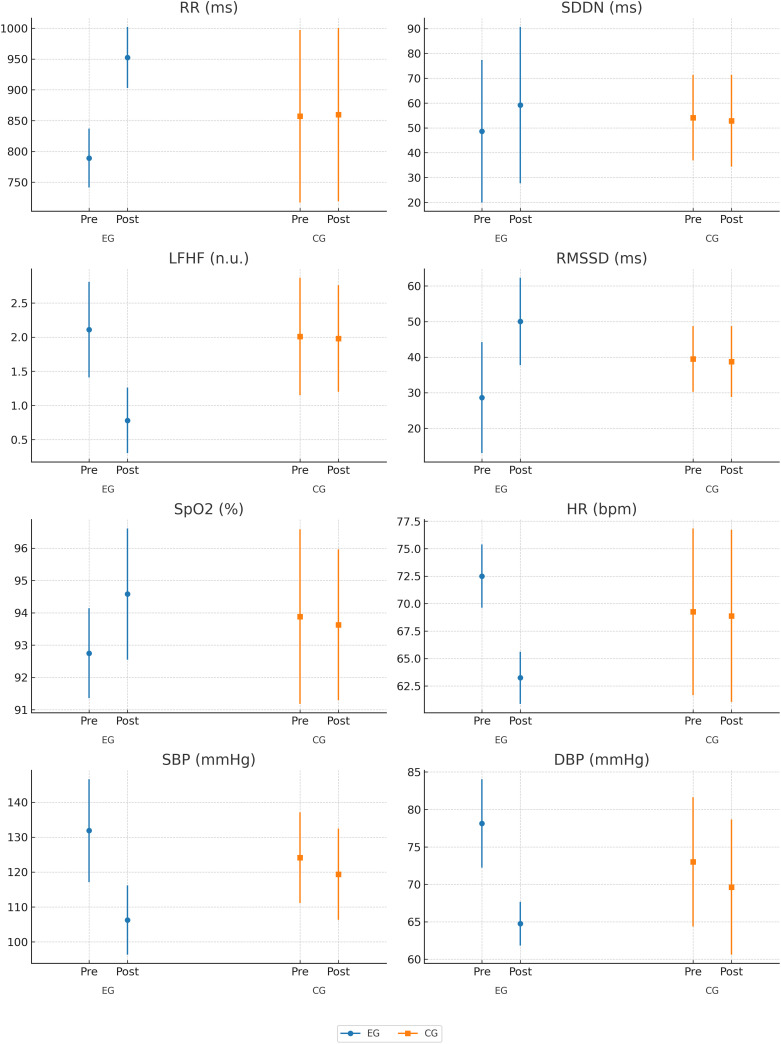
Mean ± standard deviation values of heart rate variability, oxygen saturation, and hemodynamic parameters in the experimental group (EG, blue) and control group (CG, orange), assessed at baseline (Pre) and immediately after the intervention (Post).

Results for outcomes are presented in Table 2 and Fig 2.

**Table 2:** Outcome measurements of heart rate variability, oxygen saturation, and hemodynamic parameters in the experimental group (EG) and control group (CG), expressed as mean ± standard deviation at baseline and post-intervention.

	EG	CG	Δ (95% CI)	p
RR (ms)				
Baseline	789.07 ± 47.80	857.12 ± 140.13		
Post-treatment	952.49 ± 49.62**	859.68 ± 140.62		
Difference (95% CI)	163.42 ± 44.95	2.56 ± 14.89	160.86 (124.95 to 196.77)	< 0.01
SDNN (ms)				
Baseline	48.66 ± 28.72	54.13 ± 17.25		
Post-treatment	59.17 ± 31.48**	52.87 ± 18.51		
Difference (95% CI)	10.51 ± 3.84	−1.27 ± 2.84	11.77 (8.15 to 15.39)	< 0.01
LFHF (n.u.)				
Baseline	2.11 ± 0.70	2.01 ± 0.86		
Post-treatment	0.78 ± 0.48**	1.98 ± 0.78##		
Difference (95% CI)	−1.33 ± 0.29	−0.03 ± 0.28	−1.30 (−1.60 to −0.99)	< 0.01
RMSSD (ms)				
Baseline	28.60 ± 15.63	39.49 ± 9.24		
Post-treatment	50.04 ± 12.29**	38.75 ± 9.95		
Difference (95% CI)	21.45 ± 9.18	−0.75 ± 3.62	22.19 (14.71 to 29.68)	< 0.01
SpO_2_ (%)				
Baseline	92.75 ± 1.39	93.88 ± 2.70		
Post-treatment	94.58 ± 2.03**	93.63 ± 2.33		
Difference (95% CI)	2.13 ± 0.83	−0.25 ± 2.19	2.38 (0.60 to 4.15)	< 0.01
HR (bpm)				
Baseline	72.50 ± 2.88	69.25 ± 7.59		
Post-treatment	63.25 ± 2.38**	68.88 ± 7.83		
Difference (95% CI)	−9.25 ± 2.43	−0.38 ± 2.13	−8.88 (−11.33 to −6.42)	< 0.01
SBP (mmHg)				
Baseline	131.88 ± 14.74	124.13 ± 13.00		
Post-treatment	106.25 ± 9.94**	119.38 ± 13.09#		
Difference (95% CI)	−25.63 ± 9.04	−4.75 ± 4.13	−20.88 (−28.41 to −13.34)	< 0.01
DBP (mmHg)				
Baseline	78.13 ± 5.91	73.00 ± 8.64		
Post-treatment	64.75 ± 2.92**	69.63 ± 9.02		
Difference (95% CI)	−13.38 ± 6.07	−3.38 ± 2.13	−10.00 (−14.88 to −5.12)	< 0.01

EG, Experimental group; CG, Control group; RR, Interval (R-Ri); SDNN, standard deviation of all normal-to-normal intervals; LFHF, the sympathovagal balance index as the ratio between low and high frequency power; RMSSD, the square root of the mean of the sum of squared differences between adjacent normal-to-normal intervals; SpO_2_, arterial oxygen saturation; HR, heart rate; SBP, systolic blood pressure; DBP, diastolic blood pressure.

Values are mean ± SD

*P < 0.05, **P < 0.01, post-treatment, with baseline.

#P < 0.05, ##P < 0.01, comparisons between the EG and CG groups at corresponding time points.

The repeated measures ANOVA revealed a significant increase in values along with significant group x time interactions for RR (ms). The analysis yielded a large effect size, F = 190.55, p < 0.01, ηp² = 0.93, indicating a strong and statistically significant change over time. The statistical power for this interaction was 1, suggesting that the study was fully powered to detect this effect. Similarly, for SDNN (ms), a significant group x time interaction was observed, F = 77.38, p < 0.01, ηp² = 0.85, also indicating a large effect size. The statistical power for this effect was 1, further confirming the robustness of the findings. The analysis for RMSSD (ms) also revealed a significant interaction, F = 75.57, p < 0.01, ηp² = 0.84, with a statistical power of 1. This indicates that the observed change was both large and statistically significant, suggesting a reliable effect of the intervention or condition over time. Lastly, the analysis for SpO_2_ (%) also yielded a significant interaction, F = 13.18, p < 0.01, ηp² = 0.49, with a statistical power of 0.92. While this effect size was smaller than the previous ones, it still represents a moderate to large effect with strong statistical power.

On the other hand, the repeated measures ANOVA revealed a significant decrease in values, with significant group x time interactions observed for LF/HF (n.u.). This interaction was also characterized by a large effect size, F = 171.88, p < 0.01, ηp² = 0.93, demonstrating a significant shift in the ratio over time. The statistical power for this interaction was 1, suggesting that the analysis was well-powered to detect this effect. Similarly, for HR (bpm), a significant interaction was observed, F = 130.60, p < 0.01, ηp² = 0.90. This large effect size, with a power of 1, indicates that the changes in HR over time were both highly significant and reliable. The analysis for SBP (mmHg) also yielded a significant interaction, F = 106.37, p < 0.01, ηp² = 0.88, with a statistical power of 1, supporting the robustness of the finding. Finally, the analysis for DBP (mmHg) showed a significant interaction, F = 69.15, p < 0.01, ηp² = 0.83, with a power of 1, further confirming the presence of a statistically significant effect.

In summary, the results consistently show significant group × time interactions across all measured parameters. These findings, characterized by high statistical power and large effect sizes, indicate that the intervention had a profound and reliable impact on the physiological parameters of the participants.

## 4. Discussion

The results of this study indicate that a single session of IHHE may acutely improve

HRV and blood pressure in sedentary older adults compared to a placebo application of the same therapy.

Hypoxic exposure has historically been a widely validated method in the sports field due to the benefits of simulated altitude on athletic performance. Its benefits regarding maximum oxygen consumption, angiogenesis, erythropoiesis, and metabolism subsequently led to the application of this therapy in clinical populations and sedentary individuals, due to its hormetic potential. It allows for the enhancement of mitochondrial expression and efficiency, reduction of systemic inflammation, and improvement in lactate buffering capacity and tolerance [18].

These preliminary findings suggest an improvement in HRV in the EG compared to the CG, which is in line with findings from previous studies with similar populations and interventions [8,13,19]. These results may be due to several factors. On one hand, intermittent hypoxia exposure has been shown to reduce the secretion of vasoactive substances such as dopamine, epinephrine and plasma renin activity [20]. On the other hand, intermittent hypoxia exposure attenuates the sympathetic response triggered by the stimulation of carotid chemoreceptors, producing adaptation and a decrease in sympathetic activity. Cyclical exposures to hypoxia and hyperoxia result in the activation of the solitary tract nucleus in the brainstem, producing an increase in vagal tone through parasympathetic pathways [21]. These results are highly relevant for older adults, given the importance of HRV as a biomarker and indicator of frailty, stress, cognitive function, and cardiovascular health in this population [22]. Dysfunction in autonomic control increases the risk of cardiovascular disease and is itself a risk factor [23]. HRV reflects natural fluctuations in the intervals between consecutive heartbeats, influenced by the central nervous system and serving as an indirect marker of sympathetic-parasympathetic balance. It provides insight into an individual’s capacity to regulate internal processes and adapt to physiological, psychological and environmental stressors [24].

The results of our study suggest a decrease in systolic and diastolic blood pressure values in the EG compared to the CG, highlighting the immediate antihypertensive effect of IHHE. Regarding these observed improvements, our results are consistent with previous studies [2,3,25,26]. IHHE increases nitric oxide synthesis, leading to generalized vasodilation, as well as improvements in endothelial function, oxidative stress and a reduction in cardiovascular load [26,27]. Moreover, as previously mentioned, the benefits obtained in autonomic cardiac control also have a beneficial effect on blood pressure. On one hand, increased parasympathetic activity, as well as improved carotid baroreflex sensitivity, produce a decrease in blood pressure [28]. On the other hand, IHHE reduces vascular reactivity and sympathetic activation, resulting in improved vascular responses to stress [29]. It is also worth highlighting the importance of the hypoxic recovery phase through hyperoxia, where, as demonstrated in previous studies such as that of Fratantonio et al., exposure to 30% hyperoxia promotes HIF-1 activation and transcription, thereby enhancing the previously described effects. Regarding oxygen saturation, SpO₂ showed a statistically significant but small acute increase in the experimental group compared with the control group. However, given the limited absolute magnitude of change and the narrow physiological range of this variable, its clinical relevance should be interpreted cautiously. It should be acknowledged that the most favorable physiological responses to IHHE appear to occur when relatively modest oxygen fluctuations are applied. In contrast, protocols employing larger oscillations in FiO₂ have sometimes yielded less consistent or less encouraging outcomes [26,30]. These discrepancies may be attributed to differences in populations, intervention duration and methodological heterogeneity.

This study presents a series of limitations that should be acknowledged. Although the sample size calculation was methodologically appropriate, the inclusion of both sexes increased the heterogeneity of the cohort. Additionally, the relatively small sample size limits the generalizability of the findings, potentially restricting the reliability of inferences drawn from the available data. Concerning the timing of measurements, this study evaluated only the immediate effects of IHHE, without assessing their evolution over time, thereby precluding the objective determination or clarification of potential benefits beyond the acute intervention. Furthermore, the use of techniques such as electrocardiography could have enhanced the objectivity of the HRV assessment.

Future research should involve larger sample sizes, diverse population groups, and repeated or continuous intervention protocols to validate the findings of the present study. Moreover, further investigation is required to establish the individual mean oxygen concentration values and the optimal range of oxygen variation that maximizes efficacy while ensuring safety.

## 5. Conclusion

Intermittent hypoxia–hyperoxia exposure may be associated with acute improvements in heart rate variability and reductions in systolic and diastolic blood pressure in sedentary older adults compared with a sham intervention.

## Supporting information

S1 CONSORT 2025 checklist. (DOCX)

S2 Anonymized dataset supporting the findings of this study. (XLSX)

S3 Ethics committee. (DOCX)
