# Supplementary material for: Acute effects of intermittent hypoxia–hyperoxia exposure on cardiovascular autonomic function and blood pressure in sedentary older adults: A pilot randomized controlled trial
Source: PLoS One. 2026 Jun 26;21(6):e0350802. doi: 10.1371/journal.pone.0350802 (PMC13308773; doi:10.1371/journal.pone.0350802)
Supplement: S3 — (DOCX) [file pone.0350802.s003.docx]

**S3. ETHICS COMMITTEE APPLICATION**

**INFLUENCE OF THE DIAPHRAGM ON PHYSICAL AND CARDIORESPIRATORY CONDITION IN OLDER ADULTS**

*Full English translation of the original Spanish study protocol file “COMITE HIPOXIA.docx”.*

The following research project consists of three phases:

Phase I: “Influence of one session of hypoxia-hyperoxia in older adults on muscle strength, analytical markers, respiratory function, exercise capacity, and oxygen and hemoglobin levels in peripheral musculature. Double-blind placebo randomized clinical trial.”

Phase II: “Influence of a hypoxia-hyperoxia exposure protocol in older adults on muscle strength, analytical markers, respiratory function, exercise capacity, and oxygen and hemoglobin levels in peripheral musculature. Double-blind placebo randomized clinical trial.”

The project will be carried out at the private facilities of the “Residencial Montes de Toledo” Geriatric Residence (Manzaneque).

This is an experimental study design. The investigations will be conducted in accordance with the Declaration of Helsinki and data protection legislation (Organic Law 15/1999, of 13 December).

**RESEARCH TEAM**

Arturo Ladriñán Maestro; Physiotherapist at the Montes de Toledo nursing home, professor in the degree programme at the University of Castilla-La Mancha.

Marta de la Plaza San Frutos*; PhD in Physical Activity and Sport Sciences, professor in the Physiotherapy degree programme at the European University of Madrid.

Alberto Sánchez Sierra*; PhD in Physiotherapy, professor in the Physiotherapy degree programme at the European University of Madrid.

Felipe Madruga Galán*; MD, Specialist in Geriatrics and Family and Community Medicine. Consultant Geriatrician, Virgen del Valle Hospital. SESCAM.

Lucía Ladriñán Maestro; Nurse at the National Hospital for Paraplegics. SESCAM.

**ABSTRACT**

Respiratory muscle training is an effective method that is increasingly used both in sports and health settings, employing different devices, among which threshold devices are highlighted. The objective of this study is to determine whether there is a relationship between diaphragmatic fatigue and muscle strength in the lower and upper limbs, in healthy and pathological subjects, as well as the relationship between such fatigue and other variables such as pulmonary function, maximal inspiratory pressure, functionality, heart rate variability, heart rate, and oxygen levels in muscle tissue.

The study is divided into three phases, all of them in older adults. The first phase will be carried out using a diaphragmatic fatigue protocol, the second using an inspiratory muscle warm-up protocol, and the third using an inspiratory muscle training protocol.

All phases will consist of a randomized clinical trial. In the first phase, subjects will be divided into two groups: an experimental group (performing submaximal inspiratory loads equivalent to 60% of their MIP until they are unable to establish flow in at least three maximal inspiratory efforts) and a control group (performing a protocol of two sets of 30 repetitions at 15% of MIP). In the second phase, subjects will be divided into two groups: an experimental group (performing two sets of 30 repetitions at 40% of their MIP) and a placebo group (performing two sets of 30 repetitions at 15% of their MIP). Finally, in the third phase, subjects will be divided into two groups: an experimental group (performing two sets of 30 repetitions at 40% of their MIP for 5 weeks) and a placebo group (performing two sets of 30 repetitions at 15% of their MIP for 5 weeks).

Variables will be measured including pain perception, pulmonary function, maximal inspiratory pressure, strength dynamometry, heart rate variability, heart rate, muscle oxygen and hemoglobin levels, the six-minute walk test, the Timed Up and Go test, and the 5 sit-to-stand test (FSTS).

Regarding statistical analysis, the normality of data distributions will be assessed using the Kolmogorov-Smirnov or Shapiro-Wilk test. Qualitative variables will be compared using the χ2 test or Fisher’s exact test when necessary. Quantitative variables will be compared using Student’s t test if normally distributed or the Mann-Whitney U test otherwise.

**1. INTRODUCTION**

Oxygen delivery is fundamental for energy production, recovery from exertion, and human life. During prolonged and/or intense physical exercise, greater amounts of energy are required to perform these activities, and this energy demand is directly related to a greater oxygen requirement.

Pressure changes during inhalation and exhalation occur due to the action of the respiratory musculature, which can be grouped into four groups: the diaphragm, intercostals, abdominals, and accessory muscles. The diaphragm is a double-domed muscle that originates from the central tendon and inserts into the xiphoid process, the last six ribs, and the first three lumbar vertebrae, separating the thoracic cavity from the abdominal cavity. It is innervated by the C3-C4 and C5 nerve roots, which form the right and left phrenic nerves (1). Its contraction generates 75% of inspiration and is accompanied by the scalene and intercostal muscles (2).

Intermittent hypoxia-hyperoxia exposure (IHHE) is a novel technique in which the subject, through a facial mask and a hypoxic generation device, is exposed to a respiratory environment with a reduced oxygen fraction. This is controlled by specific software connected to a pulse oximeter in order to monitor oxygen saturation at all times (3). In 2019, the Nobel Prize in Medicine highlighted that working in hypoxia, through modulation by HIF-1, can activate more than 300 genes associated with improvements in inflammatory, cognitive, cardiorespiratory, and nervous system functions, among others (4).

Various studies have demonstrated the effectiveness of IHHE in different clinical populations, including cardiac patients (5), geriatric populations (3,6), and patients with metabolic syndrome (7), first demonstrating the safety of its application (5,6,7), as well as benefits at the cardiovascular level, inflammatory status, and exercise tolerance (3,5,6,7).

This is mainly due to the effects produced by hypoxia-induced activation of HIF-1, including increased concentrations of nitric oxide, brain-derived neurotrophic factor, serotonin, AMP-activated protein kinase, and adrenomedullin (4). These changes translate into improvements in spatial-temporal memory, reductions in blood pressure, improved myocardial function, reduced inflammatory status, and improved aerobic capacity (3,6).

**2. RATIONALE**

There are numerous publications in the literature on IHHE, along with protocols reporting improvements in cardiorespiratory, cognitive, and physical conditions in clinical populations.

Our study consists of evaluating the effects of IHHE in a single session in Phase I and of a specific IHHE protocol over 3 weeks in Phase II, in order to observe the relationship between the effects of acute IHHE or 3-week IHHE and muscle strength, analytical markers, exercise capacity assessed through different tests, heart rate variability, and muscle tissue oxygen and total hemoglobin levels.

**3. HYPOTHESES**

**Phase I: Acute IHHE**

Acute IHHE in older adults could influence muscle strength, respiratory function, exercise capacity, pain, and the level of oxygenation of muscle tissue.

**Phase II: 3-week IHHE protocol**

IHHE, through a 3-week intervention protocol, could influence muscle strength, respiratory function, analytical markers, exercise capacity, and muscle tissue oxygenation levels.

**4. OBJECTIVES**

**Phase I objectives**

General objectives

• To determine whether there is a relationship between acute IHHE and muscle strength.

• To determine whether there is a relationship between acute IHHE and pulmonary function.

• To determine whether there is a relationship between acute IHHE and maximal inspiratory pressure (MIP).

• To determine whether there is a relationship between acute IHHE and exercise capacity.

Specific objectives

• To determine whether there is a relationship between acute IHHE and heart rate variability (HRV).

• To determine whether there is a relationship between acute IHHE and oxygen and total hemoglobin levels in peripheral musculature.

**Phase II objectives**

General objectives

• To determine whether there is a relationship between the 3-week IHHE protocol and muscle strength.

• To determine whether there is a relationship between the 3-week IHHE protocol and analytical markers.

• To determine whether there is a relationship between the 3-week IHHE protocol and pulmonary function.

• To determine whether there is a relationship between the 3-week IHHE protocol and maximal inspiratory pressure (MIP).

• To determine whether there is a relationship between the 3-week IHHE protocol and exercise capacity.

Specific objectives

• To determine whether there is a relationship between the 3-week IHHE protocol and heart rate variability (HRV).

• To determine whether there is a relationship between the 3-week IHHE protocol and oxygen and total hemoglobin levels in peripheral musculature.

**5. METHODOLOGY**

**Study design**

The project consists of two randomized experimental studies. The studies will be conducted after approval by the Ethics Committee. Subjects will be individually scheduled at the facilities of the “Residencial Montes de Toledo” Geriatric Residence. Before data collection begins, all participants who show interest in participating in the study and meet the selection criteria will receive an information sheet and informed consent form (see Appendices), and all questions about the development of the study will be clarified.

Inclusion criteria:

• Being over 60 years old.

Exclusion criteria:

• Having any pathology that prevents the performance of physical activity.

• Subjects without intact cognitive abilities.

• Subjects with tympanic membrane perforation or middle/inner ear pathology.

• Subjects with pulmonary hypertension, decompensated heart failure, or decompensated respiratory disease.

• Subjects who have undergone lower-limb surgery during the previous 12 months.

**Sample size (convenience calculation)**

The sample size calculation, based on previous studies (8), was performed using the G*Power 3.1.9.2 software (G*Power, University of Dusseldorf, Germany), determining an effect size of f = 0.20 with an alpha probability error adjusted to 0.05. Power (beta error) was set at 20%, with two groups and two repeated measurements over time, a correlation level between repeated measures of 0.3, and a sphericity correction of 0.5, resulting in 10 subjects in the experimental group and 10 subjects in the control group (N = 20) to detect a significant difference. A 10% loss-to-follow-up rate was estimated.

**Recruitment of the sample**

Participants will mainly be recruited from Residencia Montes de Toledo (Manzaneque), which has a total of 130 residents. Conversations have already been held with the management, who have given approval for collaboration in this study.

**Variables and assessment method**

The variables age, weight, and height will be collected, as they are important for comparison with reference values in spirometry and in the measurement of maximal inspiratory pressure and maximal expiratory pressure.

Pulmonary function: This will be measured by functional spirometric testing. The SEPAR Guidelines (2013) will be followed. An open-system pneumotachograph or spirometer will be used.

The variables obtained will be forced vital capacity (FVC), that is, the maximum volume of air expressed in milliliters that the subject is able to inhale in a forced inspiration maneuver, and FEV1, or the maximum volume exhaled in the first second measured in milliliters, which provides information on pulmonary elastic quality.

Before the test, the patient will be asked their date of birth and height, as the device will use algorithms to compare the subject with the average population for an individual with the same characteristics.

During the maneuver, the subject will be seated with the back upright, without tight clothing and without crossing the legs. During the maneuver, the back should not lift off the backrest. A nose clip will be placed to prevent air leakage.

Before starting, the investigator in charge will demonstrate the maneuver and give precise instructions on what to do. The subject will be asked first to completely empty the lungs, then to inhale as much air as possible, to make a brief pause with the lungs completely empty; the pause should be less than 1 second, and then to empty the lungs as quickly as possible until instructed to stop.

If performance defects are observed during the maneuver, the investigator will stop and correct the maneuver so that the subject learns and does not become fatigued.

For the maneuvers to be accepted, and as specified in the guidelines on acceptability criteria, the curve must have a rapid start; the maneuver must be maintained with constant exhalation without alterations or artifacts, such as coughing or closure of the glottis; and from start to finish it must last at least 6 seconds.

A minimum of three maneuvers and a maximum of eight will be performed. The best FVC and FEV1 values will be selected, even if they come from different maneuvers (8).

Muscle oxygen and hemoglobin levels: Muscle oxygen levels will be assessed using the Moxy Monitor device (Fortiori Design LLC, Hutchinson, MN, USA). This is a wireless and portable device that uses infrared spectroscopy to assess skeletal muscle oxygen saturation (SmO2) and total hemoglobin (THb). Data analysis will be performed using computer software (Moxy Software v1.5.5; Idiag, Fehraltorf, Switzerland). The measurement protocol by Contreras-Briceño et al. will be used, consisting of a 180-second measurement at rest followed by continuous measurement during exercise. The selected values will correspond to the average recorded during the last 30 seconds of each phase (9).

MIP: Inspiratory muscle strength will be assessed using maximal inspiratory pressure (MIP).

The study subject will be asked to rest for 5 minutes before performing the first maneuver. The subject will be seated, the nose will be occluded to prevent air leakage, and they will be instructed to keep the back straight. The examiner will demonstrate the maneuver before it is performed. The subject will be asked to exhale until the lungs are completely empty, hold for 1 second at maximal exhalation, and inhale as forcefully as possible. The subject will rest for 1 minute between maneuvers, up to the six maneuvers recommended in the SEPAR procedures manual.

The highest MIP value and the highest MEP value will be recorded, expressed in cmH2O, and compared with population reference values. SEPAR Procedures Manual 2003 (10).

Lower-limb/upper-limb strength dynamometry: The recovery capacity of muscle strength throughout the study will be analyzed using strength dynamometry. The subject will perform maximum-intensity isometric contractions. For lower limbs, the protocol of Hung et al. will be used: two isometric contractions of the knee extensors to determine MVC. Two contractions will be held for 5 seconds, with three minutes of rest between them to allow the phosphocreatine reserves used during this sudden maximum-intensity effort to return to baseline. Subjects will be seated on the edge of a physiotherapy table with the knee at 90 degrees of flexion. The dynamometer will be placed at the level of the malleoli to limit the lever arm and ensure the strongest possible isometric contraction. The peak force during each contraction will be sought and normalized to each subject’s weight so that it can be expressed in Newtons. Strength dynamometry will be performed using an ActiveForce 2 device (Activbody, San Diego, CA). For upper limbs, the recommendations of the American Society of Hand Therapists will be followed: the subject with the shoulder in adduction and neutral rotation, the elbow flexed at 90°, the forearm in neutral position, and the wrist positioned between 0° and 30° of extension. Three maximum handgrip measurements will be performed with the dominant hand, with one minute of rest between them, selecting the highest value. Strength dynamometry will be performed using a JAMAR device (Lafayette Instrument, Lafayette, IN, USA) (12).

Heart rate: Heart rate variability (HRV) will be analyzed using a heart rate monitor (Polar H10; Polar Electro Oy, Kempele, Finland). Cardiac electrical signals will be monitored with a chest strap for 5 minutes while the subject is supine on a table, in a quiet environment with soft lighting and laboratory temperature between 22 °C and 24 °C. Subjects will be asked not to speak or perform voluntary movements during this analysis. Data will be analyzed using Kubios HRV Stands 3.1.0 software for Windows (Biomedical Signal and Medical Imaging Analysis Group, Department of Applied Physics, University of Kuopio, Finland), and six parameters will be determined: standard deviation of the interbeat interval of normal sinus beats in milliseconds (ms), known as SDNN; low frequency (LF); high frequency (HF); sympathovagal balance index as the ratio between low- and high-frequency power (LF/HF); and Poincaré plot measures SD1 (parasympathetic activity) and SD2 (global variability) (13).

Six-minute walking test: The six-minute walk test (6MWT) is a functional cardiorespiratory test consisting of measuring the maximum distance that a subject can walk during 6 minutes (14).

Timed Up and Go test: The patient sits in a chair with arms and is instructed to stand up (start of the test and timing), walk 3 meters, and return to sit in the initial chair (end of timing). Interpretation: < 20 seconds: normal; > 20 seconds: increased risk of falling (15).

5 sit-to-stand test (FSTS): The patient sits in a chair without arms, 43-45 cm high, with arms crossed over the chest, to stand up and sit down 5 times as quickly as possible. The minimal detectable change will be 3.6 to 4.2 seconds and the minimal clinically significant difference will be 2.3 seconds. The mean execution time by age will be: 11.4 seconds for 60 to 69 years, 12.6 seconds for 70 to 79 years, and 14.8 seconds for 80 to 89 years (16,17).

**Randomization and blinding**

Two groups will be formed in Phase I, divided into Experimental Group (EG) and Control Group (CG), following randomization using Microsoft Office Excel. Two intervention devices (Energy) will be available: one will deliver hypoxia and the other normoxia. The patient will not know with which of the two devices the intervention is being performed. The assessor will be blinded, as they will not know which group each subject’s measurements belong to.

In Phase II, subjects will be divided into an Experimental Group (EG) and a Placebo Group (PG), following randomization using Microsoft Office Excel. Two intervention devices (Energy) will be available: one will deliver hypoxia and the other normoxia. The patient will not know with which of the two devices the intervention is being performed. The assessor will be blinded, as they will not know which group each subject’s measurements belong to.

**Intervention and assessment protocols in Phase I, Phase II, and Phase III**

**Phase I**

The EG will undergo one IHHE session, breathing air with an oxygen concentration (FiO2) between 10-14% for 1-5 minutes, with 1-3 minutes of rest in hyperoxia (FiO2 30-40%), for a total of 4-8 cycles depending on the acute response to hypoxia. The CG will perform a protocol of 5 cycles with an FiO2 of 21% (18).

This intervention will be supervised by health professionals, with continuous monitoring of SpO2 and HR; these data will be automatically captured by the device software. The patient’s breathing pattern will be free, provided that a minimum inspiration/expiration ratio of 1:2 is met, in order to avoid complications such as hyperventilation (dizziness). For this purpose, a physiotherapist specialized in respiratory physiotherapy will individually supervise each intervention. The intervention will last approximately 30-40 minutes at most. The device control software will have a maximum desaturation threshold preset (set at a maximum of 85%), so that if this range is reached at any time, the device will automatically begin supplying the hyperoxic cycle. External oxygen supply devices will also be available in the room and may be used in case of decompensation in any subject. Nevertheless, multiple previous studies have already performed interventions similar to ours in both clinical populations and healthy subjects without appreciable complications during their performance (3,6,7). It has been shown to be a safe and effective method (5,6,7), with multiple benefits in physical and cardiorespiratory condition (19,20).

**Phase II**

The EG will undergo 6 weeks of intervention, with alternate IHHE sessions, breathing air with an oxygen concentration (FiO2) between 10-14% for 1-5 minutes, with 1-3 minutes of rest in hyperoxia (FiO2 30-40%), for a total of 4-8 cycles depending on the acute response to hypoxia. The CG will perform a protocol of 5 cycles with an FiO2 of 21% (18).

This intervention will be supervised by health professionals, with continuous monitoring of SpO2 and HR; these data will be automatically captured by the device software. The patient’s breathing pattern will be free, provided that a minimum inspiration/expiration ratio of 1:2 is met, in order to avoid complications such as hyperventilation (dizziness). For this purpose, a physiotherapist specialized in respiratory physiotherapy will individually supervise each intervention. The intervention will last approximately 30-40 minutes at most. The device control software will have a maximum desaturation threshold preset (set at a maximum of 85%), so that if this range is reached at any time, the device will automatically begin supplying the hyperoxic cycle. External oxygen supply devices will also be available in the room and may be used in case of decompensation in any subject. Nevertheless, multiple previous studies have already performed interventions similar to the sample in both clinical populations and healthy subjects without appreciable complications during their performance (3,6,7). It has been shown to be a safe and effective method (5,6,7), with multiple benefits in physical and cardiorespiratory condition (19,20).

**Phase I assessment protocol**

A pre-intervention assessment (T0) of the variables will be performed following the previously mentioned protocols, taking approximately 30 minutes per subject. Once the intervention has been completed, all measurements will be performed again immediately (T1), again taking approximately 30 minutes.

**Phase II assessment protocol**

A pre-intervention assessment (T0) of the variables will be performed following the previously mentioned protocols, taking approximately 30 minutes per subject. Once the intervention has been completed, all measurements will be performed again immediately (T1), again taking approximately 30 minutes.

**Statistical analysis**

Qualitative variables will be expressed as absolute number and percentage, and quantitative variables as mean ± standard deviation if normally distributed or as median and interquartile range (IQR) if not. The Kolmogorov-Smirnov or Shapiro-Wilk test will be used to check the normality of data distributions. Qualitative variables will be compared using the χ2 test or Fisher’s exact test when necessary. Quantitative variables will be compared using Student’s t test if they follow a normal distribution or the Mann-Whitney U test otherwise. P values below 0.05 will be considered statistically significant.

**6. ETHICAL CONSIDERATIONS**

The project must be approved by the Ethics Committee. A patient information sheet will be provided, detailing all study considerations. An informed consent form will also be provided, which must be signed before participation and may be revoked at any time. Compliance with personal data protection legislation (Organic Law 3/2018 of 5 December on Personal Data Protection and guarantee of digital rights) will be ensured, maintaining confidentiality regarding the identity of participating subjects. The personal data of the study participants will undergo a pseudonymization process, being associated with an alphanumeric code (ID 1, ID 2, etc.) to which only the research team will have access. These data will be kept for a minimum period of 5 years (Law 14/2007, of 3 July, on Biomedical Research), after which they will be immediately destroyed. Throughout the study, the biomedical research guidelines of the Declaration of Helsinki (latest update: Fortaleza 2013) will be respected.

**7. BIBLIOGRAPHY**

1. Orozco-Levi M, Gea J. The diaphragm. Arch Bronconeumol [Internet]. 1997;33(8):399–411. Available at: http://dx.doi.org/10.1016/s0300-2896(15)30567-6

2. García-Talavera I, Díaz Lobato S, Bolado PR, Villasante C. Músculos respiratorios. Arch Bronconeumol [Internet]. 1992;28(5):239–46. Available at: http://dx.doi.org/10.1016/s0300-2896(15)31335-1

3. Behrendt T, Altorjay A-C, Bielitzki R, Behrens M, Glazachev OS, Schega L. Influence of acute and chronic intermittent hypoxic-hyperoxic exposure prior to aerobic exercise on cardiovascular risk factors in geriatric patients—a randomized controlled trial. Front Physiol [Internet]. 2022;13. Available at: http://dx.doi.org/10.3389/fphys.2022.1043536

4. Kupferschmidt K. Medicine Nobel honors work on cellular system to sense oxygen levels. Science [Internet]. 2019; Available at: http://dx.doi.org/10.1126/science.aaz7396

5. Sanz-Ayán MP, Crespo González-Calero M, Izquierdo García J, González Alcázar C, de Juan-Bagudá J, Arranz Escudero A, et al. Acondicionamiento hipóxico-hiperóxico intermitente en la rehabilitación de la insuficiencia cardiaca. REC: CardioClinics [Internet]. 2022; Available at: http://dx.doi.org/10.1016/j.rccl.2022.08.001

6. Bayer U, Likar R, Pinter G, Stettner H, Demschar S, Trummer B, et al. Effects of intermittent hypoxia-hyperoxia on mobility and perceived health in geriatric patients performing a multimodal training intervention: a randomized controlled trial. BMC Geriatr [Internet]. 2019;19(1):167. Available at: http://dx.doi.org/10.1186/s12877-019-1184-1

7. Afina AB, Oleg SG, Alexander AB, Ines D, Alexander Yu S, Nikita VV, et al. The effects of intermittent hypoxic-hyperoxic exposures on lipid profile and inflammation in patients with metabolic syndrome. Front Cardiovasc Med [Internet]. 2021;8:700826. Available at: http://dx.doi.org/10.3389/fcvm.2021.700826

8. García-Río F, Calle M, Burgos F, Casan P, Del Campo F, Galdiz JB, et al. Espirometría. Arch Bronconeumol [Internet]. 2013;49(9):388–401. Available at: http://dx.doi.org/10.1016/j.arbres.2013.04.001

9. Contreras-Briceño F, Espinosa-Ramírez M, Moya-Gallardo E, Fuentes-Kloss R, Gabrielli L, Araneda OF, et al. Intercostal muscles oxygenation and breathing pattern during exercise in competitive marathon runners. Int J Environ Res Public Health [Internet]. 2021;18(16):8287. Available at: http://dx.doi.org/10.3390/ijerph18168287

10. Neumologia DE, Toracica YC. Manual separ de procedimientos 3. Sociedad española de neumologia y cirurgia toracica (SEPAR).

11. Hung B-L, Sun C-Y, Chang N-J, Chang W-D. Effects of different kinesio-taping applications for delayed onset muscle soreness after high-intensity interval training exercise: A randomized controlled trial. Evid Based Complement Alternat Med [Internet]. 2021;2021:6676967. Available at: http://dx.doi.org/10.1155/2021/6676967

12. Roberts HC, Denison HJ, Martin HJ, Patel HP, Syddall H, Cooper C, et al. A review of the measurement of grip strength in clinical and epidemiological studies: towards a standardised approach. Age Ageing [Internet]. 2011;40(4):423–9. Available at: http://dx.doi.org/10.1093/ageing/afr051

13. Flatt AA, Esco MR. Heart rate variability stabilization in athletes: towards more convenient data acquisition. Clin Physiol Funct Imaging [Internet]. 2016;36(5):331–6. Available at: http://dx.doi.org/10.1111/cpf.12233

14. Butland RJ, Pang J, Gross ER, Woodcock AA, Geddes DM. Two-, six-, and 12-minute walking tests in respiratory disease. Br Med J (Clin Res Ed) [Internet]. 1982;284(6329):1607–8. Available at: http://dx.doi.org/10.1136/bmj.284.6329.1607

15. Hendriks S, Huisman MG, Ghignone F, Vigano A, de Liguori Carino N, Farinella E, et al. Timed up and go test and long-term survival in older adults after oncologic surgery. BMC Geriatr [Internet]. 2022;22(1):934. Available at: http://dx.doi.org/10.1186/s12877-022-03585-4

16. Schaubert KL, Bohannon RW. Reliability and validity of three strength measures obtained from community-dwelling elderly persons. J Strength Cond Res [Internet]. 2005;19(3):717–20. Available at: http://dx.doi.org/10.1519/R-15954.1

17. Bohannon RW. Reference values for the five-repetition sit-to-stand test: a descriptive meta-analysis of data from elders. Percept Mot Skills [Internet]. 2006;103(1):215–22. Available at: http://dx.doi.org/10.2466/pms.103.1.215-222

18. Behrendt T, Altorjay A-C, Bielitzki R, Behrens M, Glazachev OS, Schega L. Influence of acute and chronic intermittent hypoxic-hyperoxic exposure prior to aerobic exercise on cardiovascular risk factors in geriatric patients-a randomized controlled trial. Front Physiol [Internet]. 2022;13:1043536. Available at: http://dx.doi.org/10.3389/fphys.2022.1043536

19. Navarrete-Opazo A, Mitchell GS. Therapeutic potential of intermittent hypoxia: a matter of dose. Am J Physiol Regul Integr Comp Physiol [Internet]. 2014;307(10):R1181-97. Available at: http://dx.doi.org/10.1152/ajpregu.00208.2014

20. Lizamore CA, Kathiravel Y, Elliott J, Hellemans J, Hamlin MJ. The effect of short-term intermittent hypoxic exposure on heart rate variability in a sedentary population. Physiol Int [Internet]. 2016;103(1):75–85. Available at: http://dx.doi.org/10.1556/036.103.2016.1.7

**APPENDICES**

**APPENDIX I**

**PARTICIPANT INFORMATION SHEET AND INFORMED CONSENT**

“Influence of one session of hypoxia-hyperoxia in older adults on muscle strength, analytical markers, respiratory function, exercise capacity, and oxygen and hemoglobin levels in peripheral musculature. Double-blind placebo randomized clinical trial.”

PI: ARTURO LADRIÑÁN MAESTRO

Thank you for your interest in this study. In order for you to participate, your consent is required. Therefore, we ask you to take the necessary time to carefully read the following information and to ask any questions you consider necessary to the investigator whose contact details appear above, so that you may be truly informed.

Please be aware that, in any case, your decision to participate is entirely voluntary and that, even if you decide to participate, you may withdraw your consent at any time without any consequence for you.

**What is this study and what does it aim to achieve?**

This study/project aims to observe the influence of one session of hypoxia-hyperoxia exposure on strength, physical, functional, and cardiorespiratory condition.

Participation in the study is voluntary; no one is obliged to participate. Likewise, anyone may leave the study at any time without having to provide any explanation.

**Who is conducting this study?**

The study research team is composed of:

Arturo Ladriñán Maestro; Physiotherapist at the Montes de Toledo nursing home, professor in the degree programme at the University of Castilla-La Mancha.

Marta de la Plaza San Frutos*; PhD in Physical Activity and Sport Sciences, professor in the Physiotherapy degree programme at the European University of Madrid.

Alberto Sánchez Sierra*; PhD in Physiotherapy, professor in the Physiotherapy degree programme at the European University of Madrid.

Felipe Madruga Galán*; MD, Specialist in Geriatrics and Family and Community Medicine. Consultant Geriatrician, Virgen del Valle Hospital. SESCAM.

Lucía Ladriñán Maestro; Nurse at the National Hospital for Paraplegics. SESCAM.

**How long will the study last?**

The expected duration of this study is one month. The duration of each participant’s involvement will be approximately 1-2 hours in a single session.

**How will the study be carried out? What will my participation involve and what risks does it entail?**

A prior assessment of your physical condition (grip strength, walking capacity, functionality) and cardiorespiratory condition (diaphragm strength, pulmonary function, heart rate variability, and tissue oxygen and hemoglobin levels) will be performed, with a 2-3 minute rest between tests. Subsequently, an intervention will be performed in which, if you are in the experimental group, you will breathe through a facial mask connected to a hypoxia-hyperoxia machine. For a few minutes you will breathe air with a slightly lower oxygen concentration than ambient air, followed by another brief period with air at a higher oxygen concentration than ambient air. If you belong to the placebo group, you will breathe air with an oxygen concentration equal to ambient air, also through a facial mask connected to a hypoxia-hyperoxia machine (only the principal investigator will know which group you belong to). There is no rush when breathing, nor any time limit. Once this intervention has been completed, the previously described test battery will be performed again, with the same rest interval. These tests are simple to perform; however, you will always be supervised by a member of the research team who will guide you throughout the process and will be available for any questions. The possibility of risks or complications is extremely low, and you will be constantly monitored by healthcare professionals. Possible adverse effects may mainly include dizziness, drowsiness, or a slight feeling of shortness of breath. We will have the necessary resources to deal with any of these situations, and the intervention will of course be stopped if any of them occurs. These interventions will be carried out at the facilities of the “Residencial Montes de Toledo” Geriatric Residence.

**What data will be collected for this study?**

Personal data such as age, weight, height, health risk factors, and lifestyle habits will be collected. Regarding research data, information will be collected on strength, functional capacity, diaphragmatic strength, pulmonary function, and cardiorespiratory function.

**How is confidentiality and protection of my personal data guaranteed?**

This study involves the processing of personal data, and the investigators will guarantee confidentiality in the processing of such data at all times, complying with personal data protection regulations, in particular European Regulation 679/2016 of 27 April, the General Data Protection Regulation, as well as Organic Law 3/2018 of 5 December on Personal Data Protection and Guarantee of Digital Rights.

The personal data of subjects participating in this study will undergo a pseudonymization process, replacing their personal data with an alphanumeric code (ID 1, ID 2, etc.) to which only the principal investigator will have access.

In accordance with data protection regulations, we inform you of the following:

**DATA CONTROLLER**

The data controller is the investigator responsible for the study: Mr. Arturo Ladriñán Maestro; email: arturo.ladrinan@uclm.es; telephone: 615274176.

Only members of the research team will have access to your data, with the Principal Investigator being ultimately responsible for data processing. You should contact this person at the email address indicated above if you wish to exercise your rights regarding data protection.

**CONSENT AND PURPOSE**

Your personal data will be processed with your express consent and may only be used for additional and compatible research purposes after anonymization.

You may withdraw your consent at any time without any consequence for you.

**DATA DISCLOSURE**

The personal data collected will not be transferred without your express consent, except in cases where there is a legal obligation to do so or after anonymization of the data so that re-identification is not possible.

**DATA RETENTION**

Your personal data will be retained for a minimum period of 5 years (Law 14/2007, of 3 July, on Biomedical Research), after which they will be immediately destroyed.

**EXERCISE OF RIGHTS**

In accordance with your right to personal data protection, we inform you that you may exercise at any time your rights of access, rectification, erasure, restriction of processing, objection, and any others recognized by the General Data Protection Regulation, as well as by Organic Law 2/2018 on Personal Data Protection and guarantee of digital rights, by submitting your request to the University of Castilla-La Mancha, Albacete Campus, Plaza de la Universidad no. 2, 02071-Albacete, through the registry, through its electronic office, and via email at proteccion.datos@uclm.es.

You may also request any clarification or information regarding the exercise of these rights by contacting the Data Protection Officer of UCLM in writing at proteccion.datos@uclm.es.

Likewise, and especially if you consider that you have not obtained full satisfaction in the exercise of your rights, you may file a complaint with the national supervisory authority for data protection, the Spanish Data Protection Agency, C/ Jorge Juan, 6 – 28001 Madrid, or at www.aepd.es.

More information about the protection of your personal data is available at https://www.uclm.es/legal/informacion-legal/proteccion-datos

**THIS IS THE END OF THE INFORMATION SHEET FOR YOU TO CONSIDER WHETHER OR NOT TO ACCEPT PARTICIPATION IN THE STUDY.**

We remind you that you may request any clarification or ask any question to ensure that you have all the information you need to make your decision.

If you decide to participate, we ask you to complete and sign the following “Informed Consent” form, indicating that you accept and consent to participate in the study after having received all the information.

**INFORMED CONSENT**

I (name of the participant/patient or representative): ______________________________

On my own behalf (mark if applicable)

On behalf of another person (mark if applicable).

Name of the person I represent: ______________________________

And, acknowledging that I have taken into account their previously expressed wishes or objections regarding this study,

I confirm that I have read the information sheet that has been provided to me. I state that I have understood its contents and that I have been given the opportunity to ask the questions I considered necessary in order to understand it properly. I therefore express my free and informed willingness to participate voluntarily in the study, acknowledge that I have been given a copy of this consent, and expressly consent, by my signature, to the processing of my personal data for the previously mentioned purposes in relation to the management and execution of the research project.

In ___________________ on ____ of _______ 20__

| Name and surname of participant/representative   Signature | Name and surname of investigator   Signature |
| --- | --- |
|  |  |

**RIGHT OF REVOCATION**

(If you wish to exercise your right to withdraw your consent)

I (name of the participant/patient or representative): ______________________________

On my own behalf (mark if applicable)

On behalf of another person (mark if applicable).

Name of the person I represent: ______________________________

And, acknowledging that I have taken into account their previously expressed wishes or objections regarding this study,

I revoke the informed consent previously granted as of today …… of ………… of ……….. and do not wish to continue in the study, considering it terminated as of the date described above. I also acknowledge that I have been given a copy of this revocation.

| Name and surname of participant/representative   Signature | Name and surname of investigator   Signature |
| --- | --- |
|  |  |

**APPENDIX II**

**PARTICIPANT INFORMATION SHEET AND INFORMED CONSENT**

“Influence of a hypoxia-hyperoxia exposure protocol in older adults on muscle strength, analytical markers, respiratory function, exercise capacity, and oxygen and hemoglobin levels in peripheral musculature. Double-blind placebo randomized clinical trial.”

PI: ARTURO LADRIÑÁN MAESTRO

Thank you for your interest in this study. In order for you to participate, your consent is required. Therefore, we ask you to take the necessary time to carefully read the following information and to ask any questions you consider necessary to the investigator whose contact details appear above, so that you may be truly informed.

Please be aware that, in any case, your decision to participate is entirely voluntary and that, even if you decide to participate, you may withdraw your consent at any time without any consequence for you.

**What is this study and what does it aim to achieve?**

This study/project aims to observe the influence of a 6-week hypoxia-hyperoxia exposure protocol on strength, physical, functional, and cardiorespiratory condition. Participation in the study is voluntary; no one is obliged to participate. Likewise, anyone may leave the study at any time without having to provide any explanation.

**Who is conducting this study?**

The study research team is composed of:

Arturo Ladriñán Maestro; Physiotherapist at the Montes de Toledo nursing home, professor in the degree programme at the University of Castilla-La Mancha.

Marta de la Plaza San Frutos*; PhD in Physical Activity and Sport Sciences, professor in the Physiotherapy degree programme at the European University of Madrid.

Alberto Sánchez Sierra*; PhD in Physiotherapy, professor in the Physiotherapy degree programme at the European University of Madrid.

Felipe Madruga Galán*; MD, Specialist in Geriatrics and Family and Community Medicine. Consultant Geriatrician, Virgen del Valle Hospital. SESCAM.

Lucía Ladriñán Maestro; Nurse at the National Hospital for Paraplegics. SESCAM.

**How long will the study last?**

The expected duration of this study is 3 months. The duration of each participant’s involvement will be approximately 1 hour, on alternate days, for 6 weeks.

**How will the study be carried out? What will my participation involve and what risks does it entail?**

A prior assessment of your physical condition (grip strength, walking capacity, functionality) and cardiorespiratory condition (diaphragm strength, pulmonary function, heart rate variability, and tissue oxygen and hemoglobin levels) will be performed, with a 2-3 minute rest between tests. In addition, a blood test will be performed to assess your general condition. Subsequently, an intervention will be performed in which, if you are in the experimental group, you will breathe through a facial mask connected to a hypoxia-hyperoxia machine. For a few minutes you will breathe air with a slightly lower oxygen concentration than ambient air, followed by another brief period with air at a higher oxygen concentration than ambient air. If you belong to the placebo group, you will breathe air with an oxygen concentration equal to ambient air, also through a facial mask connected to a hypoxia-hyperoxia machine (only the principal investigator will know which group you belong to). There is no rush when breathing, nor any time limit. Once this intervention has been completed, the previously described test battery will be performed again, with the same rest interval. These tests are simple to perform; however, you will always be supervised by a member of the research team who will guide you throughout the process and will be available for any questions. The possibility of risks or complications is extremely low, and you will be constantly monitored by healthcare professionals. Possible adverse effects may mainly include dizziness, drowsiness, or a slight feeling of shortness of breath. We will have the necessary resources to deal with any of these situations, and the intervention will of course be stopped if any of them occurs. These interventions will be carried out at the facilities of the “Residencial Montes de Toledo” Geriatric Residence.

**What data will be collected for this study?**

Personal data such as age, weight, height, health risk factors, and lifestyle habits will be collected. Regarding research data, information will be collected on strength, functional capacity, diaphragmatic strength, analytical markers, pulmonary function, and cardiorespiratory function.

**How is confidentiality and protection of my personal data guaranteed?**

This study involves the processing of personal data, and the investigators will guarantee confidentiality in the processing of such data at all times, complying with personal data protection regulations, in particular European Regulation 679/2016 of 27 April, the General Data Protection Regulation, as well as Organic Law 3/2018 of 5 December on Personal Data Protection and Guarantee of Digital Rights.

The personal data of subjects participating in this study will undergo a pseudonymization process, replacing their personal data with an alphanumeric code (ID 1, ID 2, etc.) to which only the principal investigator will have access.

In accordance with data protection regulations, we inform you of the following:

**DATA CONTROLLER**

The data controller is the investigator responsible for the study: Mr. Arturo Ladriñán Maestro; email: arturo.ladrinan@uclm.es; telephone: 615274176.

Only members of the research team will have access to your data, with the Principal Investigator being ultimately responsible for data processing. You should contact this person at the email address indicated above if you wish to exercise your rights regarding data protection.

**CONSENT AND PURPOSE**

Your personal data will be processed with your express consent and may only be used for additional and compatible research purposes after anonymization.

You may withdraw your consent at any time without any consequence for you.

**DATA DISCLOSURE**

The personal data collected will not be transferred without your express consent, except in cases where there is a legal obligation to do so or after anonymization of the data so that re-identification is not possible.

**DATA RETENTION**

Your personal data will be retained for a minimum period of 5 years (Law 14/2007, of 3 July, on Biomedical Research), after which they will be immediately destroyed.

**EXERCISE OF RIGHTS**

In accordance with your right to personal data protection, we inform you that you may exercise at any time your rights of access, rectification, erasure, restriction of processing, objection, and any others recognized by the General Data Protection Regulation, as well as by Organic Law 2/2018 on Personal Data Protection and guarantee of digital rights, by submitting your request to the University of Castilla-La Mancha, Albacete Campus, Plaza de la Universidad no. 2, 02071-Albacete, through the registry, through its electronic office, and via email at proteccion.datos@uclm.es.

You may also request any clarification or information regarding the exercise of these rights by contacting the Data Protection Officer of UCLM in writing at proteccion.datos@uclm.es.

Likewise, and especially if you consider that you have not obtained full satisfaction in the exercise of your rights, you may file a complaint with the national supervisory authority for data protection, the Spanish Data Protection Agency, C/ Jorge Juan, 6 – 28001 Madrid, or at www.aepd.es.

More information about the protection of your personal data is available at https://www.uclm.es/legal/informacion-legal/proteccion-datos

**THIS IS THE END OF THE INFORMATION SHEET FOR YOU TO CONSIDER WHETHER OR NOT TO ACCEPT PARTICIPATION IN THE STUDY.**

We remind you that you may request any clarification or ask any question to ensure that you have all the information you need to make your decision.

If you decide to participate, we ask you to complete and sign the following “Informed Consent” form, indicating that you accept and consent to participate in the study after having received all the information.

**INFORMED CONSENT**

I (name of the participant/patient or representative): ______________________________

On my own behalf (mark if applicable)

On behalf of another person (mark if applicable).

Name of the person I represent: ______________________________

And, acknowledging that I have taken into account their previously expressed wishes or objections regarding this study,

I confirm that I have read the information sheet that has been provided to me. I state that I have understood its contents and that I have been given the opportunity to ask the questions I considered necessary in order to understand it properly. I therefore express my free and informed willingness to participate voluntarily in the study, acknowledge that I have been given a copy of this consent, and expressly consent, by my signature, to the processing of my personal data for the previously mentioned purposes in relation to the management and execution of the research project.

In ___________________ on ____ of _______ 20__

| Name and surname of participant/representative   Signature | Name and surname of investigator   Signature |
| --- | --- |
|  |  |

**RIGHT OF REVOCATION**

(If you wish to exercise your right to withdraw your consent)

I (name of the participant/patient or representative): ______________________________

On my own behalf (mark if applicable)

On behalf of another person (mark if applicable).

Name of the person I represent: ______________________________

And, acknowledging that I have taken into account their previously expressed wishes or objections regarding this study,

I revoke the informed consent previously granted as of today …… of ………… of ……….. and do not wish to continue in the study, considering it terminated as of the date described above. I also acknowledge that I have been given a copy of this revocation.

| Name and surname of participant/representative   Signature | Name and surname of investigator   Signature |
| --- | --- |
|  |  |
