# Supplementary material for: Acute effects of intermittent hypoxia–hyperoxia exposure on cardiovascular autonomic function and blood pressure in sedentary older adults: A pilot randomized controlled trial
Source: PLoS One. 2026 Jun 26;21(6):e0350802. doi: 10.1371/journal.pone.0350802 (PMC13308773; doi:10.1371/journal.pone.0350802)
Supplement: Attachment — Submitted filename: RESPONSE TO REVIEWERS PLOS.docx [file pone.0350802.s005.docx]

**RESPONSE TO REVIEWERS**

We sincerely thank the Editor and the Reviewers for their thoughtful comments and constructive suggestions, which have helped us to improve the clarity and scientific quality of the manuscript. All comments have been carefully considered and addressed point by point below. Corresponding revisions in the manuscript are highlighted in color for ease of reference.

**REVIEWER 1**

The authors would like to thank the reviewers for their helpful comments and suggestions and for their assistance in improving the quality of the manuscript. The comments from each reviewer are addressed individually below. Changes within the manuscript are highlighted (yellow). We believe that our manuscript is improved in the light of the suggested changes.

1. **Title: due to small sample size, the paper should be entilted as pilot**

We thank the reviewer for this valuable suggestion and agree that, given the exploratory nature and limited sample size of the present study, explicitly identifying the trial as a pilot study improves the transparency and interpretation of the findings.

Accordingly, the title has been revised to include the term *“A Pilot Randomized Controlled Trial.”*

2. **Introduction should be shortened**

We appreciate this suggestion and have revised the Introduction to improve conciseness and focus. Redundant background information and overly detailed physiological explanations were reduced, while preserving the rationale and scientific context necessary to support the study objective.

3. **Methods: XXXXXXXXXXXXXXX should be removed**

We thank the reviewer for identifying this issue. The placeholder text corresponded to anonymized information included during blinded peer review and has now been removed/replaced with the appropriate final institutional and registry details in the revised manuscript.

4. **Methods: it should be added who was blinded**

We appreciate this important methodological observation. The manuscript has been revised to provide a more detailed description of the blinding procedures implemented in the trial.

Specifically, we now clarify that participants and outcome assessors were blinded to group allocation, whereas the therapist administering the intervention could not be blinded due to the nature of the intervention but was not involved in assessments or statistical analyses.

5. **Methods: primary end point shoul dbe clearly stated**

We thank the reviewer for this recommendation. To improve methodological clarity and comply with reporting standards for randomized controlled trials, the primary and secondary endpoints have now been explicitly specified in the Outcomes section.

6. **Discussiom: due to paucity of data conclusions should be softened**

We agree with the reviewer that the conclusions should be interpreted cautiously given the pilot nature of the study and the limited sample size. Accordingly, the Discussion and Conclusion sections have been revised to adopt a more conservative interpretation of the findings and to better reflect the preliminary nature of the results.
